# Modelling Duchenne muscular dystrophy in *MYOD1-*converted urine-derived cells treated with 3-deazaneplanocin A hydrochloride

**DOI:** 10.1038/s41598-019-40421-z

**Published:** 2019-03-07

**Authors:** Hotake Takizawa, Yuko Hara, Yoshitaka Mizobe, Taisuke Ohno, Sadafumi Suzuki, Ken Inoue, Eri Takeshita, Yuko Shimizu-Motohashi, Akihiko Ishiyama, Mikio Hoshino, Hirofumi Komaki, Shin’ichi Takeda, Yoshitsugu Aoki

**Affiliations:** 1 Department of Molecular Therapy, National Institute of Neuroscience, National Center of Neurology and Psychiatry, Tokyo, Japan; 2 Department of Mental Retardation and Birth Defect Research, National Institute of Neuroscience, National Center of Neurology and Psychiatry, Tokyo, Japan; 3 Department of Child Neurology, National Center Hospital, National Center of Neurology and Psychiatry, Tokyo, Japan; 4 Department of Biochemistry & Cellular Biology, National Institute of Neuroscience, National Center of Neurology and Psychiatry, Tokyo, Japan; 5 Department of NCNP Brain Physiology and Pathology, Graduate School of Medical and Dental Sciences, Tokyo Medical and Dental University, Tokyo, Japan

**Keywords:** Molecular biology, Cell biology

## Abstract

Duchenne muscular dystrophy (DMD) is a severe muscle disorder characterised by mutations in the *DMD* gene. Recently, we have completed a phase I study in Japan based on systemic administration of the morpholino antisense that is amenable to exon-53 skipping, successfully. However, to achieve the effective treatment of DMD, *in vitro* assays on patient muscle cells to screen drugs and patient eligibility before clinical trials are indispensable. Here, we report a novel *MYOD1-*converted, urine-derived cells (UDCs) as a novel DMD muscle cell model. We discovered that 3-deazaneplanocin A hydrochloride, a histone methyltransferase inhibitor, could significantly promote *MYOGENIN* expression and myotube differentiation. We also demonstrated that our system, based on UDCs from DMD patients, could be used successfully to evaluate exon-skipping drugs targeting *DMD* exons including 44, 50, 51, and 55. This new autologous UDC-based disease modelling could lead to the application of precision medicine for various muscle diseases.

## Introduction

Duchenne muscular dystrophy (DMD) is a severe muscle disorder characterized by mutations in the *DMD* gene that mainly disrupt the reading frame, leading to the absence of functional protein^1^. Exon-skipping using short antisense oligonucleotides (ASOs) is a promising therapy for DMD, and this aims to convert the more severe DMD phenotype into the milder Becker muscular dystrophy phenotype by altering pre-mRNA splicing and restoring the open reading frame^2^. Recently, we completed a phase I study based on systemic administration of the phosphorodiamidate morpholino oligomer (PMO) NS-065/NCNP-01, which induces exon-53 skipping in DMD, to achieve a highly favourable safety profile, promising pharmacokinetics, and efficacy^3^. However, to achieve economical and efficient treatment options for DMD, *in vitro* assays using patient muscle cells are indispensable for screening new drugs and patient eligibility before clinical trials, in addition to biomarkers that reflect the efficacy of ASO-based treatments during clinical trials.

Recently, Antoury *et al*. reported that using mRNA isolated from DMD patient urine, they could successfully detect exon deletions in the *DMD* gene and confirmed exon-skipping activity after treatment with eteplirsen, an ASO that was granted accelerated approval by the U.S. Food and Drug Administration in September of 2016^4^. Their findings of ASO-dependent exon-skipping activity in urine provides the first non-invasive evaluation of ASO efficacy during a clinical trial.

In addition, we previously reported an *in vitro* assay system based on fluorescence-activated cell sorting (FACS)-isolated *MYOD1*-converted fibroblasts to determine patient eligibility before clinical trials^5^. However, one limitation of this *in vitro* assay is the requirement for an invasive skin biopsy. Therefore, it is necessary to establish a non-invasive *in vitro* assay using human urine-derived cells (UDCs), reported to be a mixed population of either renal epithelial or uroepithelial cells expressing most mesenchymal stem cell and peripheral cell markers^6,7^. Kim *et al*. demonstrated the direct-reprogramming of UDCs derived from limb-girdle muscular dystrophy and DMD patients to myogenic cells expressing MYOD1 using a lentiviral vector. For this, they choose a cell population with a rice-grain appearance expressing *Desmin*, and it took up to 4–5 weeks of cell culture until *MYOD1*-converted UDCs expressed sufficient myosin heavy chain (MyHC) or DYSTROPHIN^8^. Recently, Falzarano *et al*. reported the attempted evaluation of exon-skipping by RT-PCR in *MYOD1*-converted UDCs from one patient suffering from DMD, but they failed to detect the restoration of DYSTROPHIN by immunoblotting^9^. The most significant limitation of previous reports is the insufficient direct-reprogramming efficiency of UDCs into myotubes as an ideal muscle model of DMD.

The purpose of this study was to establish a new non-invasive *in vitro* assay system capable of efficiently evaluating exon-skipping at the mRNA and protein levels using patient-derived UDCs. To achieve this, we developed a retroviral doxycycline (Dox)-regulated inducible *MYOD1* expression system, which enables us to select cells using puromycin instead of FACS and to regulate cell proliferation/differentiation after *MYOD1* transduction. Furthermore, we discovered that 3-deazaneplanocin A hydrochloride (DZNep), a histone methyltransferase inhibitor, could significantly promote late muscle regulatory factors including *MYOGENIN*, leading to well-differentiated myotubes in a short amount of time. Using this new system, we successfully and effectively converted UDCs into myotubes. We also demonstrated exon-skipping using the new assay with UDCs from DMD patients with various mutations in a hotspot region. Moreover, we show that the assay can efficiently identify ASO sequences that specifically skip the targeted exon, leading to an efficient ASO screening and precision medicine platform for muscle diseases that can be targeted by exon-skipping.

## Results

### UDCs comprise a population of mesenchymal-like cells expressing CD90/CD73 markers

**Figure 1:**
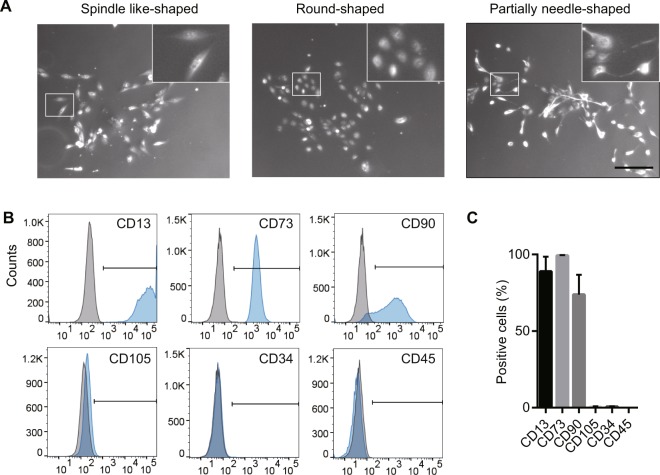
Characteristics of urine-derived cells (UDCs). (**A**) Representative pictures of UDCs are shown including spindle like- (left), round- (middle), and partially needle like (right)-shaped. Scale bar denotes 200 μm. Inset: Magnified image of the area in white rectangle. (**B**,**C**) Flow cytometric analysis of cell surface markers in UDCs. Expression of CD90 and CD105 (mesenchymal stem cell markers), CD73 (mesenchymal stem cell and epithelial cell marker), CD13 (epithelial cell marker), and CD34 and CD45 (hematopoietic lineage markers) was analysed; n = 4, for each marker. Data are expressed as mean ± SEM. Isotype-matched control antibodies (grey) were included in every experiment to define the threshold for each specific signal and to establish the appropriate gate for positive cells.

To characterize the cell population of proliferative UDCs, we collected these cells from four healthy individuals (8-, 13-, 33-, and 38-year-old males) according to the steps described in previous reports with some modifications^10^. No bacterial contamination was found in any culture samples. The characterization of UDCs has been shown in Fig. 1 and Supplementary Fig. S1. Specifically, UDCs that could form colonies had various morphologies, but those with three types of morphology, specifically spindle like-, round-, and partially needle-shaped, could proliferate well after the 4^th^ passage of primary culture (Fig. 1A). To test proliferate ability of UDCs, we isolated single colonies with these morphologies using sterilized plastic cylinders. As a result, these cells could proliferate over 6 weeks, and spindle like- and round-shaped UDCs, in particular, had marked proliferative ability (Supplementary Fig. S1A). To determine the origin of UDCs, total RNA was extracted at the 4^th^ passage after primary culture, and RT-PCR was performed targeting kidney and urinary tract-specific mRNA (Supplementary Fig. S1B). These cells were found to express γ-glutamyl transferase^11^ and kidney-specific protein (KSP)-Cadherin 16^12^, which are specific for the kidney. Moreover, they expressed cytokeratin 8^13^, which is expressed on epithelial cells. However, *AQP1*, specific for renal proximal tubular cells, was expressed only in partially needle-shaped UDCs. These mRNA expression patterns suggest that UDCs with high proliferative ability originated from the renal epithelial or uroepithelial cells including renal proximal tubular cells, which is consistent with earlier studies^6,7,14,15^. Immunophenotyping of surface antigens by flow cytometry was also performed on UDCs after the 4^th^ passage in primary culture. As a result, UDCs strongly expressed CD90 (a mesenchymal stem cell marker), CD73 (a mesenchymal stem cell and epithelial cell marker), and CD13 (an epithelial cell marker), but were negative for CD34 and CD45 (hematopoietic lineage markers) as previously reported^8,9,16^; however, they only marginally expressed CD105 (a mesenchymal stem cell marker; Fig. 1B,C).

### DZNep, a histone methyltransferase inhibitor, significantly promotes *MYOD1*-converted UDC myotube formation

**Figure 2:**
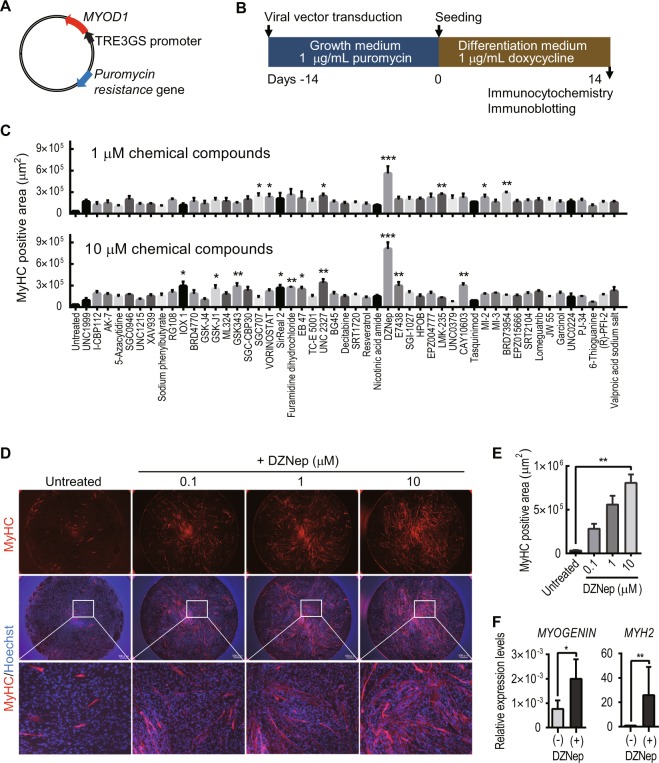
*MYOD1* and 3-deazaneplanocin A hydrochloride promote the direct-reprogramming of urine-derived cells into myotubes. (**A**) Schema of the retroviral vector with the *MYOD1* and *puromycin resistance* genes. The TRE3GS promoter is activated in the presence of doxycycline. (**B**) Schematic diagram of the transduction of the viral vector. (**C**) Results of drug screening using a chemical library (Sigma; S990043-EPI1). Representative data are shown. The area of myosin heavy chain (MyHC)-positivity was determined by fluorescence microscopy at 14^th^ day after differentiation. Urine-derived cells (UDCs) were pre-treated with various chemical compounds for initial 3 days after differentiation (final concentrations = 0.1, 1, and 10 μM). The Kruskal-Wallis test followed by a Dunn’s post hoc test was used for statistical analysis; *P < 0.05, **P < 0.01, ***P < 0.001. Data are expressed as mean ± SEM. (**D**) Representative images of immunocytochemistry for MyHC (red; scale bar, 500 μm) at 14^th^ day after differentiation. UDCs were treated with 3-deazaneplanocin A hydrochloride (DZNep) for initial 3 days after differentiation. Blue; Hoechst staining. (**E**) MyHC positive area at 14^th^ day after differentiation with and without DZNep pre-treatment was calculated. The Kruskal-Wallis test followed by a Dunn’s post hoc test was used for statistical analysis; **P < 0.01. Data are expressed as mean ± SEM. (**F**) qRT-PCR analysis for *MYOGENIN* expression on the 7^th^ day and myosin heavy chain-2 (MYH2) expression on the 14^th^ day after differentiation. UDCs were pre-treated with 1 μM DZNep for initial 3 days after differentiation; n = 4, for each. The Mann-Whitney test was used for statistical analysis.

**Figure 3:**
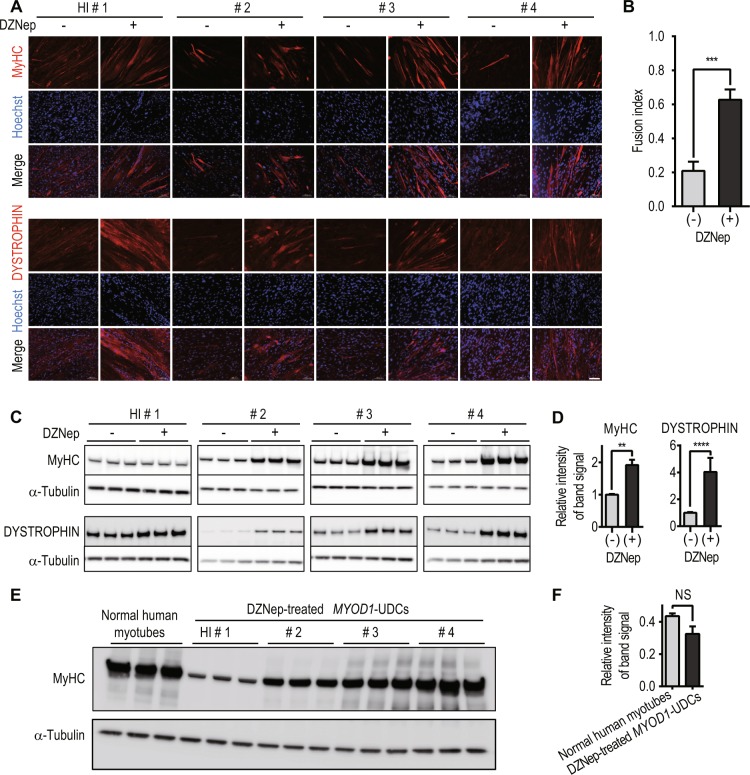
Successful myotube differentiation of 3-deazaneplanocin A hydrochloride (DZNep)-treated *MYOD1*-urine-derived cells (UDCs) derived from healthy individuals. (**A**) Representative images of immunocytochemistry for MyHC (upper) and DYSTROPHIN (lower) in *MYOD1*-UDCs from healthy children and adults 14 days after differentiation. HI: healthy individual. #1: 8-year-old male; #2: 13-year-old male; #3: 33-year-old male; #4: 38-year-old male. Scale bar: 100 μm. (**B**) Comparison of fusion index with and without DZNep treatment. The fusion index was calculated as a percentage of nuclei inside the MyHC positive myotubes using randomly selected three pictures from four healthy individuals respectively. The Mann-Whitney test was used for comparisons; ***P < 0.001. Data are expressed as mean ± SEM. (**C**) Immunoblotting analysis for MyHC (upper) and DYSTROPHIN (lower). An anti-α-tubulin antibody was used as a loading control respectively. (**D**) The relative intensities of the immunoblotting bands normalized to α-Tubulin expression was compared between *MYOD1*-UDCs with and without DZNep treatment shown in figure C using Image Lab 6.0 (Bio-Rad). The Mann-Whitney test was used for comparisons; **P < 0.01, ****P < 0.0001. Data are expressed as mean ± SEM. (**E**) Immunoblotting analysis of MyHC in normal human myotubes 1 week after differentiation (Lonza; CC-2580) and DZNep-treated *MYOD1*-UDCs. (**F**) The relative intensities of the bands normalized to α-Tubulin expression was compared between normal human myotubes and DZNep-treated *MYOD1*-UDCs shown in figure E using Image Lab 6.0. The Mann-Whitney test was used for comparisons; NS: not significant.

Next, to establish directly-reprogrammed myotubes from UDCs, we created a retroviral vector with the Dox-inducible *MYOD1* gene and a *puromycin resistance* gene enabling us to select cells using puromycin instead of FACS (Fig. 2A). This vector can regulate cell proliferation or differentiation after *MYOD1* transduction because *MYOD1* can be induced at any time by adding Dox to the culture medium. UDCs from healthy individuals were infected with the *MYOD1*-retroviral vector at a multiplicity of infection (MOI) of 200 in growth medium, and subsequently, MYOD1-positive cells were selected by adding 1 μg/mL puromycin to the medium. *MYOD1*-transduced UDCs were differentiated by changing the growth medium to differentiation medium containing 1 μg/mL Dox (Fig. 2B). Immunocytochemistry analysis 2 weeks after differentiation showed that the expression of MYOD1 was detected in many *MYOD1*-converted UDCs (*MYOD1*-UDCs). However, the expression of *MYOGENIN*, initiated by *MYOD1*^17^ and with a crucial role in the terminal differentiation of muscle cells^18–20^, was rarely detected in *MYOD1*-UDCs compared to that in *MYOD1*-converted fibroblasts (*MYOD1*-Fibs) (Supplementary Fig. S2A,B). Quantitative RT-PCR analysis of *MYOD1* and *MYOGENIN* expression after differentiation also revealed significant discrepancy, specifically high expression of *MYOD1* and low expression of *MYOGENIN* in *MYOD1*-UDCs (Supplementary Fig. S2C). This discrepancy was not corrected by various MOIs of the retroviral vector, ranging from 100 to 100,000 (Supplementary Fig. S2D,E). We hypothesised that the discrepancy between expression levels of *MYOD1* and *MYOGENIN* was due to the strong epigenetic suppression of the latter. To overcome epigenetic suppression, we screened various epigenetic drugs using a chemical library purchased from Sigma in *MYOD1*-UDCs and found that DZNep significantly promoted the formation of MyHC-positive, multinucleated myotubes from *MYOD1*-UDCs in a dose-dependent manner at 14^th^ day after differentiation (Fig. 2C–E and Supplementary Fig. S3). After treatment with 1 μM DZNep for initial 3 days after differentiation, *MYOGENIN* and *myosin heavy chain 2 (MYH2)* expression levels on the 7^th^ and 14^th^ day respectively were upregulated significantly (Fig. 2F). Using *MYOD1*-UDCs from two male children and two healthy adult men, we confirmed that total MyHC and DYSTROPHIN expression of multinucleated myotubes were significantly enhanced by treatment with DZNep based on immunocytochemistry and immunoblotting (Fig. 3A–D, and Supplementary Fig. S5A–D). We also clearly detected α-ACTININ, DESMIN, β-Dystroglycan, and sarcomere structures in DZNep-treated *MYOD1*-UDCs by immunocytochemistry, suggesting they were well-differentiated (Supplementary Fig. S4). There were no significant differences in the expression levels of MyHC between DZNep-treated *MYOD1*-UDCs and normal human myotubes 1 week after differentiation evaluated by immunoblotting for MyHC using Image Lab 6.0 (Bio-Rad) (Fig. 3E,F, and Supplementary Fig. S5E). These data show that inhibition of histone methyltransferase by DZNep could significantly promote myotube differentiation.

### Successful *in vitro* evaluation of exon-skipping in DZNep-treated *MYOD1*-UDCs derived from DMD patients with an exon deletion in the hotspot region

**Figure 4:**
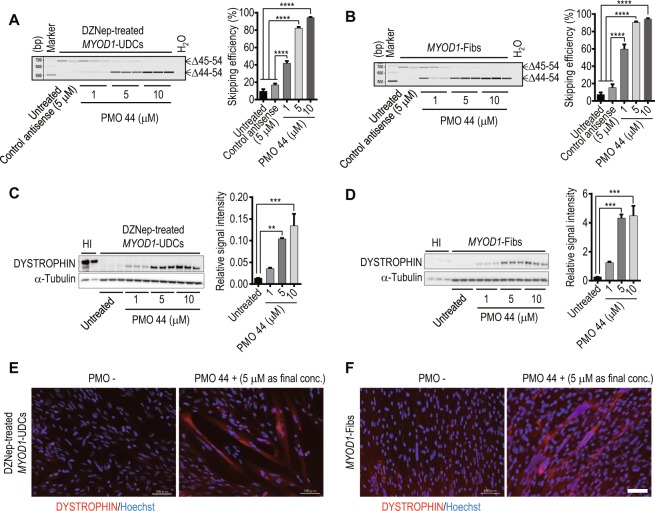
Successful evaluation of exon-skipping in urine-derived cells (UDCs) from patients with exon 45–54 deletions. RT-PCR analysis of *DYSTROPHIN* after phosphorodiamidate morpholino oligomer (PMO) treatment in (**A**) 3-deazaneplanocin A hydrochloride (DZNep)-treated *MYOD1*-UDCs and (**B**) *MYOD1*-converted fibroblasts (*MYOD1*- Fibs) derived from a Duchenne muscular dystrophy (DMD) patient with an exon 45–54 deletion. DZNep-treated *MYOD1*-UDCs and *MYOD1*- Fibs were also treated with control antisense at 5 μM as controls. The upper bands are unskipped products (Δ45–54) that remain out of the reading frame. The lower bands are exon 44-skipped products (Δ44–54) that restore the open reading frame. Skipping efficiency was calculated as (exon 44-skipped transcript molarity)/(native + exon 44-skipped transcript molarity (marked with arrows)) ×100% using MultiNA. One-way ANOVA followed by Bonferroni’s post hoc test was used to compare the skipping efficiencies; n = 3, for each group; ****P < 0.0001. Data are expressed as mean ± SEM. PMO 44: PMO for skipping exon 44. (**C**,**D**) Immunoblotting for DYSTROPHIN in DZNep-treated *MYOD1*-UDCs and *MYOD1*-Fibs from the DMD patient after PMO 44 treatment. For DYSTROPHIN detection, anti-dystrophin AB15277 (against C-terminal) was used. The relative intensities of the bands normalized to α-Tubulin expression were compared in patient-derived cells with and without PMO treatment by performing a one-way ANOVA followed by Bonferroni’s post hoc test; n = 3, for each. **P < 0.01, ***P < 0.001. HI: healthy individual. (**E,F**) Immunocytochemistry for DYSTROPHIN in the DZNep-treated *MYOD1*-UDCs and *MYOD1*-Fibs after PMO 44 treatment. Representative pictures are shown. Scale bar: 100 μm.

To determine whether we could evaluate exon-skipping using UDCs as well as fibroblasts, we collected skin and urine, and cultured skin fibroblasts and UDCs from two DMD patients. The first patient was a 6-year-old male with an exon 45–54 deletion in the *DMD* gene diagnosed by the multiplex ligation-dependent probe amplification (MLPA) method, a reliable quantitative method to detect deletions and duplications in all 79 exons of the *DMD* gene. The second patient was an 11-year-old male with an exon 45 deletion diagnosed by MLPA and sequencing of the bordering area (DMD-1 and 2 in Table 1). Their open reading frames were restored by exon 44 skipping. Next, DZNep-treated *MYOD1*-UDCs and *MYOD1*-Fibs were transfected with an antisense PMO using Endo-Porter, on the 7^th^ day after differentiation. On the 14^th^ day after differentiation, we could clearly demonstrate exon-skipping in a dose-dependent manner at the mRNA and protein level in DZNep-treated *MYOD1*-UDCs and *MYOD1*-Fibs from both DMD patients by RT-PCR and immunoblotting, respectively (Fig. 4A–D, Supplementary Fig. S6A–D). Immunocytochemistry also revealed the recovery of DYSTROPHIN after treatment with PMO (Fig. 4E,F, Supplementary Fig. S6E,F).

**Table 1:** Characteristics of DMD patients.

Subjects	Age (years)	Sample types	DMD exon deletion	Target exon for skipping	Forward primer (5′ to 3′)	Reverse primer (5′ to 3′)
DMD-1	6	Skin & Urine	45–54	44	ATGCTCCTGACCTCTGTGCT	GACTGCATCATCGGAACCTT
DMD-2	11	Skin & Urine	45	44	GCTCAGGTCGGATTGACATT	GGGCAACTCTTCCACCAGTA
DMD-3	5	Urine	50	51	ACAACCGGATGTGGAAGAGA	GCCTCTTGATTGCTGGTCTT
DMD-4	13	Urine	51	50	ACAACCGGATGTGGAAGAGA	GCCTCTTGATTGCTGGTCTT
DMD-5	10	Urine	54	55	CGGGCTTGGACAGAACTTAC	GACTGCATCATCGGAACCTT

DMD exon deletions were restored in-frame by skipping the target exon. Primers were used for RT-PCR to evaluate exon-skipping efficiency. DMD: Duchenne muscular dystrophy.

**Figure 5:**
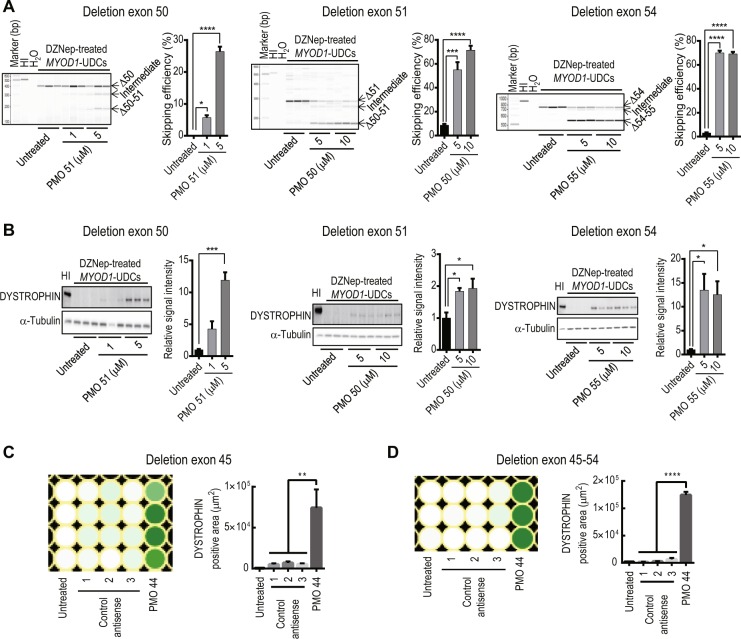
Successful evaluation of exon-skipping using urine-derived cells (UDCs) from patients with various exon deletions in a hotspot region. (**A**) RT-PCR analysis of *DYSTROPHIN* after phosphorodiamidate morpholino oligomer (PMO) treatment in 3-deazaneplanocin A hydrochloride (DZNep)-treated *MYOD1*-UDCs derived from Duchenne muscular dystrophy (DMD) patients with exon 50, 51, and 54 deletions are shown. Exon 50, 51, or 54 deletions were restored in-frame by skipping exons 51, 50, or 55, respectively. Exon-skipping efficiency was calculated as (exon-skipped transcript molarity)/(native + intermediate + exon-skipped transcript molarity (marked with arrows)) × 100% using MultiNA. One-way ANOVA followed by Bonferroni’s post hoc test was used to compare the skipping efficiency between patient-derived DZNep-treated *MYOD1*-UDCs; n = 3, each. *P < 0.05, ***P < 0.001, ****P < 0.0001. Data are expressed as mean ± SEM. HI: healthy individual. PMO51, 50, and 55: PMOs for skipping exon 51, 50, and 55, respectively. (**B**) Immunoblotting for DYSTROPHIN in DZNep-treated *MYOD1*-UDCs from DMD patients with exon 50, 51, or 54 deletions (same patients as shown in (**A**)) after PMO treatment. The relative intensities of the bands normalized to α-Tubulin expression was compared in patient-derived cells with and without PMO treatment by performing one-way ANOVA followed by Bonferroni’s post hoc test; n = 3, for each; *P < 0.05, ***P < 0.001. The relative signal intensities of the untreated were set as 1.0. (**C**,**D**) Heatmaps of immunocytochemistry for DYSTROPHIN after PMO treatment in DZNep-treated *MYOD1*-UDCs from DMD patients (DMD-1 and 2 in Table 1). Deletion of exon 45 or exon 45–54 restored the open reading frame based on the exon-skipping of exon 44. The signal intensity was quantified using a BZ-X800 microscope and a BZ-H4XI image cytometry software (KEYENCE), 1 week after ASO transfection on a 96-well plate. One-way ANOVA followed by Bonferroni’s post hoc test was used for comparison; n = 3–4, for each. **P < 0.01, ****P < 0.0001.

To assess whether our new non-invasive *in vitro* assay could be applied to other DMD patients with various mutations in the *DMD* gene in a hotspot region, specifically from exon 45 to 55 where DMD-causing deletion mutations accumulate^21–23^, we collected urine and cultured primary UDCs from three DMD patients with exon 50, 51, or 54 deletions (DMD-3, 4, and 5 in Table 1). The reading frames of these exon deletions are restored by skipping exon 51, 50, or 55, respectively. The sequences and positions of the PMOs used for this are described in Supplementary Table S1. As a result, we were able to clearly evaluate exon-skipping in a dose-dependent manner at the mRNA (Fig. 5A) and protein (Fig. 5B) level using DZNep-treated *MYOD1*-UDCs from these DMD patients. These data indicate that our new assay has a wide range for evaluating exon-skipping.

### The *in vitro* evaluation of an ASO sequence using DZNep-treated *MYOD1*-UDCs is highly recommended before *in vivo* studies

The screening of ASO sequences is normally performed using immortalized human myoblast cell lines including human rhabdomyosarcoma (RD) cells, but ideally, it would be better to perform this assay using primary muscle cells derived from DMD patients before proceeding to *in vivo* studies. This is because DYSTROPHIN restoration is essential as a surrogate biomarker to predict the benefits of exon-skipping. In other words, we cannot evaluate the recovery of DYSTROPHIN levels using RD cell lines or muscle cells derived from healthy individuals because they express this protein endogenously.

To test whether our *in vitro* assay could determine the most suitable ASO sequence at the protein level, we used DZNep-treated *MYOD1*-UDCs derived from two DMD patients with a deletion in exon 45 or 45–54 (DMD-1 and 2 in Table 1), amenable to exon 44 skipping, with various PMOs targeting different exons. To establish highly efficient image based *in vitro* sequence screening assay, we quantified the signal intensities of DYSTROPHIN using the BZ-X800 microscope and the BZ-H4XI image cytometry software, 1 week after ASO transfection on a 96-well plate. As a result, we confirmed significantly higher fluorescent signals in *MYOD1*-UDCs treated with PMO 44 compared to that in *MYOD1*-UDCs treated with control antisense oligonucleotides. These results suggest that our new assay could screen optimal ASO sequences targeted various exons very efficiently in *MYOD1*-UDCs using a 96-well plate before a clinical study, leading to precision medicine for DMD (Fig. 5C,D, Supplementary Fig. S7).

## Discussion

Here, we report the direct-reprogramming of human UDCs into myotubes using a Dox-inducible *MYOD1* retroviral vector. We also discovered that an epigenetic modification using DZNep could significantly promote the cell lineage conversion of UDCs into myotubes. Well-differentiated DZNep-treated *MYOD1*-UDCs are suggested to be an ideal *in vitro* model of DMD muscle cells. Using these cells, it is possible to investigate pathophysiology and screen for ASO efficacy. UDCs can be obtained non-invasively and repeatedly in a short period and with low expense, and thus we expect that this newly established *in vitro* assay could be adapted to a wide range of studies regardless of age, sex, and muscle disease type.

*MYOD1* is regarded as a myogenic regulatory factor that regulates myogenic differentiation^24^. Following the discovery of *MYOD1*-mediated direct-reprogramming in fibroblasts^25^, Weintraub *et al*. explored its ability to convert a diverse set of starting cell types to myogenic cells including adipocytes, neuroblastomas, and liver cells^26^. In contrast, *MYOGENIN* has a crucial role in the terminal differentiation of committed muscle cells^18–20^; however, *MYOD1* cannot compensate for its absence during skeletal muscle differentiation^27^. We failed to detect one mesenchymal stem cell marker, CD105, a membrane glycoprotein that forms a TGF-β receptor complex on cell membranes^28^ in UDCs, indicating that stemness was decreased in our UDCs compared to those previously reported^6,9,16,29^. In recent years, it was revealed that the regulation of developmental genes with dynamic expression patterns is not driven only by transcription factor networks, but also by the epigenome^30,31^. Therefore, we hypothesized that the reprogramming of terminally differentiated UDCs by epigenetic treatment could be sufficient to achieve lineage conversion — actually, our method using DZNep, a histone methyltransferase inhibitor, significantly enhanced *MYOGENIN* expression in *MYOD1-*UDCs, leading to multinucleated myotubes that express sufficient amounts of muscle-related proteins including MyHC, α-ACTININ, and DYSTROPHIN. However, an understanding of the epigenetic regulation required for efficient cell lineage conversion is still unclear and warrants additional research.

Our results suggest that exon-skipping efficiency and restoration of DYSTROPHIN in DMD patient-derived *MYOD1*-UDCs might be variable. For example, DYSTROPHIN expression after treatment with 1 μM of PMO 44 was increased 2.6- and 8.4-fold compared to expression without treatment based on immunoblotting using DZNep-treated *MYOD1*-UDCs derived from DMD-1 and DMD-2 samples, respectively, as shown in Fig. 4C and Supplementary Fig. S6C. There are several possible causes of this difference. First, our UDCs were heterogeneous and consisted of a mixed population of primary renal/uroepithelial cells, as previously reported^6,7,14,15^. Second, differentiation levels of DZNep-treated *MYOD1*-UDCs might be variable because we detected different expression levels of MyHC and DYSTROPHIN in these cells derived from healthy individuals. Finally, our new *in vitro* assay might reflect exon-skipping efficiency *in vivo*; however, a discrepancy in skipping efficiency between cell culture and *in vivo* systems was reported during the selection of ASOs targeting exons of the gene encoding dog *dystrophin*^32^. Thus, in future work, it must be determined if exon-skipping efficiency using this new *in vitro* assay correlates with exon-skipping efficiency *in vivo* by performing a clinical trial.

In conclusion, we successfully established a new UDC-based *in vitro* assay that could be used to screen drugs and evaluate patient eligibility before clinical trials, leading to improved precision medicine for various muscle diseases.

## Methods

### Ethics Statement

The Ethics Committee of the National Center of Neurology and Psychiatry approved our study, approval ID: A2017-018, A2018-029, and all individuals gave informed consent before providing urine or skin samples. All experiments were performed in accordance with relevant guidelines and regulations.

### Isolation and primary culture of UDCs

Urine samples were collected using sterilized plastic bottles. UDCs were isolated according to a previously published protocol^10^ with some modifications. Briefly, entire urine samples were centrifuged at 400 × *g* for 10 min at room temperature. The cell pellet was resuspended in primary medium composed of a 1:1 mix of high glucose DMEM without sodium pyruvate (GE Healthcare, Logan, UT) and Ham’s F-12 Nutrient Mix (Thermo Fisher Scientific, Waltham, MA) supplemented with REGM SingleQuots (Lonza, Basel, Switzerland), 10% tetracycline-free foetal bovine serum (FBS; Clontech), 1% penicillin/streptomycin (P/S), and 0.5 μg/mL amphotericin B. Cells were seeded in gelatine-coated plates and cultured at 37 °C with humidity and 5% CO_2_ for 3 days. On day 4, the medium was replaced with growth medium consisting of a 1:1 mix of REGM Bullet Kit (Lonza) and high glucose DMEM supplemented with 15% tetracycline-free FBS, 0.5% Glutamax (Thermo Fisher Scientific), 0.5% nonessential amino acids (Thermo Fisher Scientific), and 2.5 ng/mL of fibroblast growth factor-basic (bFGF; Sigma, St Louis, USA), PDGF-AB (Peprotech, Rocky Hill, NJ), EGF (Peprotech), and 1% P/S. The growth medium was changed every other day.

### Isolation and primary culture of human fibroblasts

Primary skin fibroblasts were generated from skin biopsies using a disposable biopsy punch (Kai industries co., Gifu, Japan) from the upper inner arm, after the administration of local infiltrative anaesthesia using 1% lidocaine. The fibroblasts were grown in high glucose DMEM with GlutaMAX-I (Thermo Fisher Scientific) supplemented with 10% tetracycline-free FBS, 1% P/S, and bFGF at 37 °C with humidity and 5% CO_2_. The culture medium was exchanged every 2–3 days.

### Flow cytometry

Flow cytometric evaluation of primary UDCs (4^th^ passage) was performed with fluorescent-conjugated mouse monoclonal antibodies (Supplementary Table S2). Primary UDCs were trypsinized and washed with growth medium. Cells were centrifuged at 350 × *g* for 5 min, collected, and suspended in phosphate buffered saline (PBS) with 2% FBS. The antibodies were added at optimal concentrations (Supplementary Table S2), and incubated for 20 min on ice in the dark. Cells were centrifuged, collected, resuspended in PBS with 2% FBS, passed through a 70-μm filter, and analysed using a FACSAria Fusion (BD Biosciences, San Jose, CA). Data were analysed using FlowJo v10 software (BD Biosciences).

### Retroviral construct and transduction

The pRetroX-*MTOD1*-IRES-ZsGreen1 expression vector was kindly provided by Dr. Takashi Saito. The coding region of *MYOD1* was amplified by PCR and cloned into pRetroX-TetOne-Puro Vector (Clontech) by performing an In-Fusion cloning reaction (Clontech). For retroviral production, GP2-293 packaging cells (Clontech) were seeded in collagen-coated plates, cultured in DMEM with 10% FBS, and co-transfected with the *MYOD1*-inserted pRetroX-TetOne-Puro vectors and pVZV-G capsid vectors using Xfect transfection reagent (Clontech). The viral supernatant was collected 24–48 h after co-transfection. For *MYOD1* transduction, UDCs or fibroblasts were seeded at 3,000–5,000 cells/cm^2^ and infected with retrovirus at an MOI of 200, 24 h after seeding, by adding polybrene (Sigma) at 8 μg/mL. After a 24-h incubation at 37 °C with humidity and 5% CO_2_, the culture medium was replaced with fresh growth medium containing 1 μg/mL puromycin to select *MYOD1*-transduced cells.

### Myogenic differentiation of MYOD1-transduced cells

To differentiate *MYOD1*-transduced UDCs into myotubes, cells were plated in collagen I-coated wells at a density of 3.5 × 10^4^ cells/cm^2^; 24 h later, the growth medium was changed to differentiation medium composed of high glucose DMEM with GlutaMAX-I (Thermo Fisher Scientific), 5% horse serum, ITS Liquid Media Supplement (Sigma), and 1 μg/mL Dox.

To differentiate *MYOD1*-transduced fibroblasts into myogenic cells, the cells were plated in wells coated with collagen I at a density of 2.8 × 10^4^ cells/cm^2^; 24 h later, growth medium was changed to differentiation medium composed of high glucose DMEM with GlutaMAX-I, 2% horse serum, and ITS Liquid Media Supplement with 1 μg/mL Dox.

### Antisense PMO transfection

PMOs targeting exon 50, 51, and 55, listed in Supplementary Table S1, were synthesized by Nippon Shinyaku Co., Ltd. (Kyoto, Japan). We transfected antisense PMOs at 1, 5, or 10 μM (final concentration) into *MYOD1*-converted UDCs or *MYOD1*-converted fibroblasts from DMD patients on the 7^th^ day after differentiation using the Endo-Porter transfection reagent (Gene Tools, Philomath, OR, USA). After 72-h incubation with PMOs, the medium was changed to fresh differentiation medium free of PMOs.

### RNA analysis

For RT-PCR, cells were lysed, and RNA was harvested using the RNeasy kit (Qiagen, Hilden, Germany). One microgram of total RNA was used as a template for RT-PCR with cDNA reverse transcription kits (Applied Biosystems, Warrington, UK). For one RT-PCR reaction, 1 μL of cDNA template was mixed with 14.9 μL of water, 0.2 μL of 10 μM forward primer, 0.2 µL of 10 μM reverse primer, 1.6 μL of 2.5 mM dNTPs, 2 μL of 10 × Ex Taq Buffer, and 0.1 μL Ex Taq HS from the Ex Taq Hot Start Version kit (Takara Bio, Shiga, Japan). The primer sequences are shown in Supplementary Table S3. The cycling conditions were 95 °C for 4 min, 35 cycles of 94 °C for 30 s, 55–60 °C for 30 s, 72 °C for 1 min, and finally 72 °C for 4 min. 18s RNA (Thermo Fisher Scientific) was used as an internal control. PCR products were detected using agarose gel or MultiNA, a microchip electrophoresis system (Shimadzu, Kyoto, Japan). Exon-skipping efficiency (%) was calculated as (exon-skipped transcript molarity)/(native + intermediate + exon-skipped transcript molarity) × 100% using MultiNA.

For quantitative PCR, cDNA synthesized from 1 μg of total RNA and predesigned FAM-MGB-conjugated TaqMan probes for target genes (Supplementary Table S4) were used. *EIF2B1* was used as an internal housekeeping reference, because it expressed stably in fibroblasts and UDCs during myogenic differentiation evaluated using TaqMan Array 96 Well Plate, Endogenous control (Applied Biosystems).

### Protein extraction and immunoblotting analysis

Total protein was extracted from cultured cells using RIPA buffer containing protease inhibitors (Roche, Indianapolis, IN, USA). The lysates were sonicated on ice and centrifuged at 14,000 × *g* for 15 min at 4 °C. The supernatant was collected, and protein concentrations were determined using a BCA protein assay kit (Thermo Fisher Scientific). After mixing with NuPAGE LDS Sample Buffer (Thermo Fisher Scientific), cell lysates were denatured at 70 °C for 10 min, electrophoresed using NuPAGE Novex Tris-Acetate Gel 3–8% (Invitrogen) at 150 V for 75 min, and then transferred to PVDF membranes. The membranes were incubated with primary antibodies, which was followed by incubation with secondary antibody using the iBind Flex Western Device (Thermo Fisher Scientific). The following primary antibodies were used: rabbit anti-dystrophin (1:500, Abcam, Cambridge, UK; ab15277), mouse anti-myosin heavy chain (1:200, R&D, Minneapolis, USA; MAB4470), and mouse anti-α-tubulin (1:1000, Sigma; T6199). Histofine Simple Stain MAX-PO (1:100, NICHIREI BIOSCIENCE INC., Tokyo, Japan; 424151) was used as a secondary antibody. Proteins were detected using the ECL Prime Western Blotting Detection Reagent (GE Healthcare, UK; RPN2232) and a ChemiDoc MP Imaging System (Bio-Rad, Hercules, CA, USA). Data were analysed using Image Lab 6.0 (Bio-Rad).

### Immunofluorescence microscopy

For immunofluorescence analysis, cells were washed with PBS, fixed in 4% paraformaldehyde for 10 min at 4 °C, and subsequently permeabilized in 0.1% triton-X (MP Biomedicals, USA) for 10 min at RT. Cells were blocked with 10% goat serum for 15 min at 37 °C. Primary antibody incubations were performed overnight at 4 °C. Cells were then washed with PBS and incubated with secondary antibodies for 30 min at RT. Plates were imaged using a fluorescent microscope (BZ-9000 or BZ-X800, KEYENCE, Osaka, Japan) and BZ-X Analyzer or BZ-X800 Analyzer (KEYENCE). The following primary antibodies were used: mouse anti-MYOD1 (1:200, Santa Cruz; sc-32758), mouse anti-myogenin (1:200, Santa Cruz; sc-12732), mouse anti-dystrophin (1:30, Leica, NCL-DYS1), mouse anti-myosin heavy chain (1:50, R&D; MAB4470), mouse anti-α-Actinin (1:1000, Sigma; A7811), mouse anti-desmin (1:800, Sigma; D1033), and mouse anti-β-dystroglycan (1:100, Leica, NCL-b-DG). Alexa Fluor 546 goat anti-mouse IgG (H + L; 1:300, Invitrogen) or anti-mouse IgG, Dylight 488 (Vector Laboratories, USA; DK-2488) were used as secondary antibodies. Nuclei were stained with Hoechst 33342 (1:10,000; Thermo Fisher Scientific; H3570).

### Screening of chemical compounds that promote direct myogenic reprogramming

A chemical library for epigenetics research containing 80 compounds was purchased from Sigma (S990043-EPI1). *MYOD1*-transduced UDCs from a healthy individual were seeded at a density of 35,000 cells/cm^2^ on collagen-coated 96-well plates and cultured in growth medium; 24 h later, the medium was changed to differentiation medium with 1 μg/mL Dox, and each chemical compound was added at 0.1, 1, and 10 μM. Three days later, media were removed and replaced with new differentiation medium with 1 μg/mL Dox. Eleven days later, the expression of MyHC was examined by immunofluorescence staining and measured based on fluorescence intensity using a fluorescent microscope (BZ-9000, KEYENCE) and BZ-X Analyzer (KEYENCE). To calculate the MyHC positive area, we selected four fields from each well randomly and analysed under the same condition.

### Statistical analysis

All data are presented as mean ± SEM. GraphPad Prism 6 (GraphPad Software, Inc., La Jolla, CA) was used to perform data analysis with a Mann–Whitney test for two-sample comparison, and a Kruskal-Wallis test followed by the Dunn’s post hoc test or one-way analysis of variance (ANOVA) followed by a Bonferroni’s post hoc test for more than two groups. P-values < 0.05 was considered statistically significant.

## Supplementary information

Supplementary Dataset 1

## Data Availability

All data generated or analysed during the present study are included in this published article (and its Supplementary Information Files).
