# Publisher Correction: Modelling Duchenne muscular dystrophy in *MYOD1*-converted urine-derived cells treated with 3-deazaneplanocin A hydrochloride

**DOI:** 10.1038/s41598-020-59351-2

**Published:** 2020-02-07

**Authors:** Hotake Takizawa, Yuko Hara, Yoshitaka Mizobe, Taisuke Ohno, Sadafumi Suzuki, Ken Inoue, Eri Takeshita, Yuko Shimizu-Motohashi, Akihiko Ishiyama, Mikio Hoshino, Hirofumi Komaki, Shin’ichi Takeda, Yoshitsugu Aoki

**Affiliations:** 1 Department of Molecular Therapy, National Institute of Neuroscience, National Center of Neurology and Psychiatry, Tokyo, Japan; 2 Department of Mental Retardation and Birth Defect Research, National Institute of Neuroscience, National Center of Neurology and Psychiatry, Tokyo, Japan; 3 Department of Child Neurology, National Center Hospital, National Center of Neurology and Psychiatry, Tokyo, Japan; 4 Department of Biochemistry & Cellular Biology, National Institute of Neuroscience, National Center of Neurology and Psychiatry, Tokyo, Japan; 5 Department of NCNP Brain Physiology and Pathology, Graduate School of Medical and Dental Sciences, Tokyo Medical and Dental University, Tokyo, Japan

Correction to: *Scientific Reports* 10.1038/s41598-019-40421-z, published online 07 March 2019

In the Supplementary Information file originally published with this Article, Supplementary Figures (Figures S1–7) and Supplementary Tables (Tables S1–4) were omitted. These errors have been corrected in the Supplementary Information that now accompanies the Article.

