# Supplementary material for: Modelling Duchenne muscular dystrophy in MYOD1-converted urine-derived cells treated with 3-deazaneplanocin A hydrochloride
Source: Sci Rep. 2019 Mar 7;9:3807. doi: 10.1038/s41598-019-40421-z (PMC6405839; doi:10.1038/s41598-019-40421-z)
Supplement: Supplementary file 1 — Supplementary Dataset 1 [file 41598_2019_40421_MOESM1_ESM.pdf]

Modelling Duchenne muscular dystrophy in *MYOD1*-converted urine-derived cells treated with 3-deazaneplanocin A hydrochloride

Hotake Takizawa[1,5], Yuko Hara[1], Yoshitaka Mizobe[1], Taisuke Ohno[1], Sadafumi Suzuki[2], Ken Inoue[2], Eri Takeshita[3], Yuko Shimizu-Motohashi[3], Akihiko Ishiyama[3], Mikio Hoshino[4,5], Hirofumi Komaki[3], Shin'ichi Takeda[1], Yoshitsugu Aoki[1,5*]

1 Department of Molecular Therapy, National Institute of Neuroscience, National Center of Neurology and Psychiatry, Tokyo, Japan.

2 Department of Mental Retardation and Birth Defect Research, National Institute of Neuroscience, National Center of Neurology and Psychiatry, Tokyo, Japan.

3 Department of Child Neurology, National Center Hospital, National Center of Neurology and Psychiatry, Tokyo, Japan.

4 Department of Biochemistry & Cellular Biology, National Institute of Neuroscience, National Center of Neurology and Psychiatry, Tokyo, Japan.

5 Department of NCNP Brain Physiology and Pathology, Graduate School of Medical and Dental Sciences, Tokyo Medical and Dental University, Tokyo, Japan.

* To whom correspondence should be addressed: Yoshitsugu Aoki. Tel: [+81-42-346-1720]; Fax: [+81-42- 346-1750]; Email: [tsugu56@ncnp.go.jp]

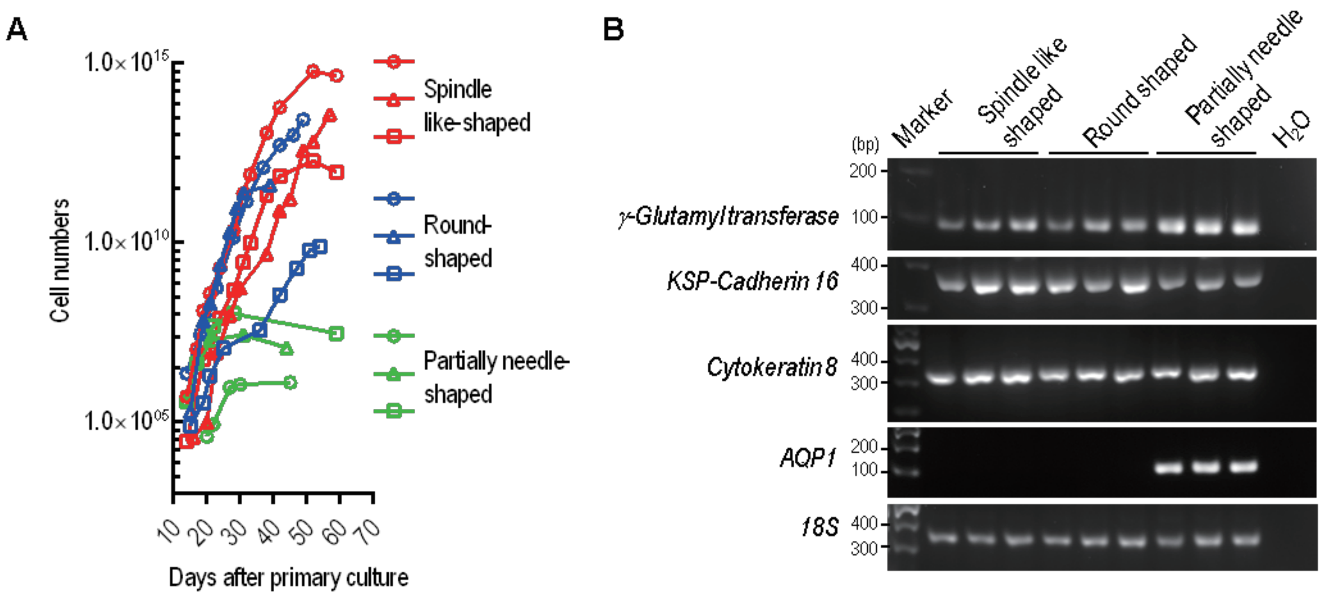

Supplementary Figure S1. Characteristics of urine-derived cells (UDCs). (A) Cell proliferation curves of UDCs with the three morphologies. (B) mRNA expression in UDCs with the three morphologies (three colonies each), as detected by RT-PCR targeting renal-specific genes.

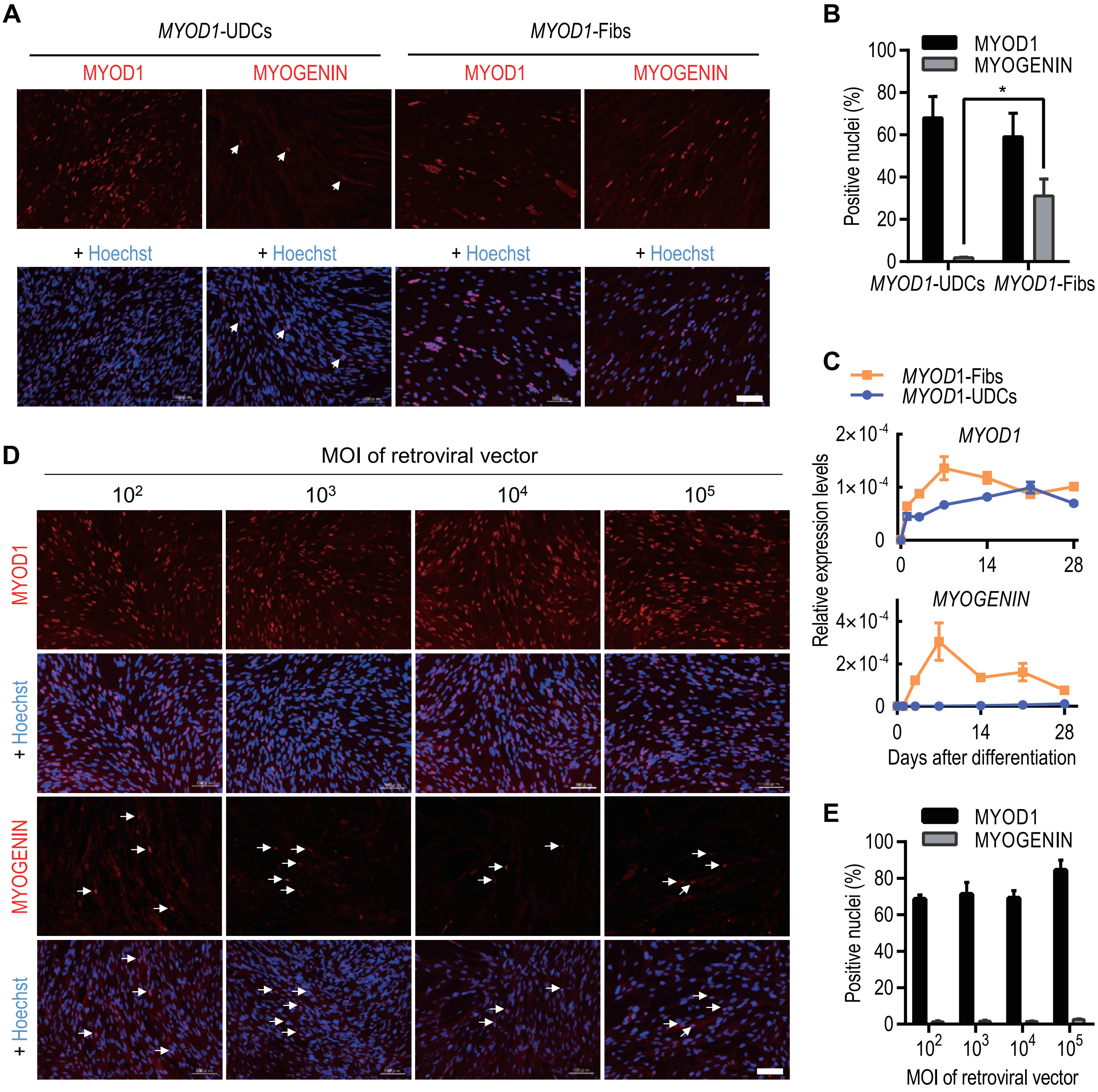

Supplementary Figure S2. Expression levels of *MYOD1* and *MYOGENIN* in *MYOD1*-converted urine-derived cells (UDCs) and *MYOD1*-converted fibroblasts. (A) Immunocytochemistry on *MYOD1*-UDCs and *MYOD1*-Fibs 2 weeks after differentiation. Representative images are shown. Arrows show MYOGENIN-positive nuclei in *MYOD1*-UDCs. Scale bar: 100 µm. (B) The percentage of MYOD1- and MYOGENIN-positive nuclei were compared between *MYOD1*-UDCs and *MYOD1*-Fibs shown in figure A. Three pictures were randomly selected from *MYOD1*-UDCs and *MYOD1*-Fibs respectively. (C) qRT-PCR analysis of *MYOD1* (upper) and *MYOGENIN* (lower) expression levels relative to *EIF2B1*, an internal control, after differentiation; n = 3, for each. Data are expressed as mean ± SEM. (D) Immunocytochemistry on *MYOD1*-UDCs after retroviral infection at various MOIs, 2 weeks after differentiation. Representative images are shown. Arrows show MYOGENIN-positive nuclei. MOI: multiplicity of infection. (E) The percentage of MYOD1- and MYOGENIN-positive nuclei were compared between *MYOD1*-UDCs infected at various MOIs shown in figure D. Three pictures were randomly selected from various MOIs respectively.

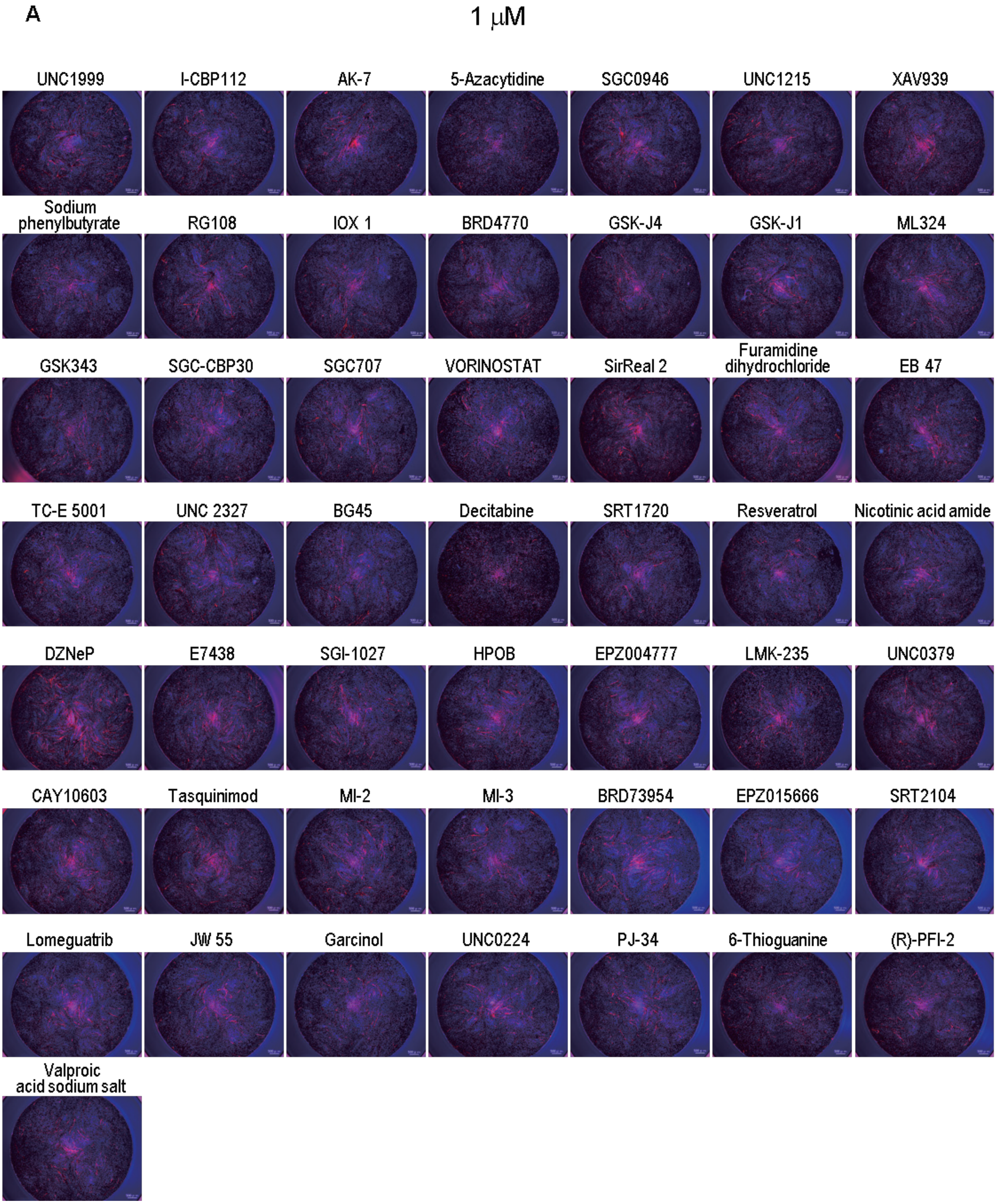
A
1 μM
UNC1999
I-CBP112
AK-7
5-Azacytidine
SGC0946
UNC1215
XAV939
Sodium phenylbutyrate
RG108
IOX 1
BRD4770
GSK-J4
GSK-J1
ML324
GSK343
SGC-CBP30
SGC707
VORINOSTAT
SirReal 2
Furamidine dihydrochloride
EB 47
TC-E 5001
UNC 2327
BG45
Decitabine
SRT1720
Resveratrol
Nicotinic acid amide
DZNeP
E7438
SGI-1027
HPOB
EPZ004777
LMK-235
UNC0379
CAY10603
Tasquinimod
MI-2
MI-3
BRD73954
EPZ015666
SRT2104
Lomeguatrib
JW 55
Garcinol
UNC0224
PJ-34
6-Thioguanine
(R)-PFI-2
Valproic acid sodium salt

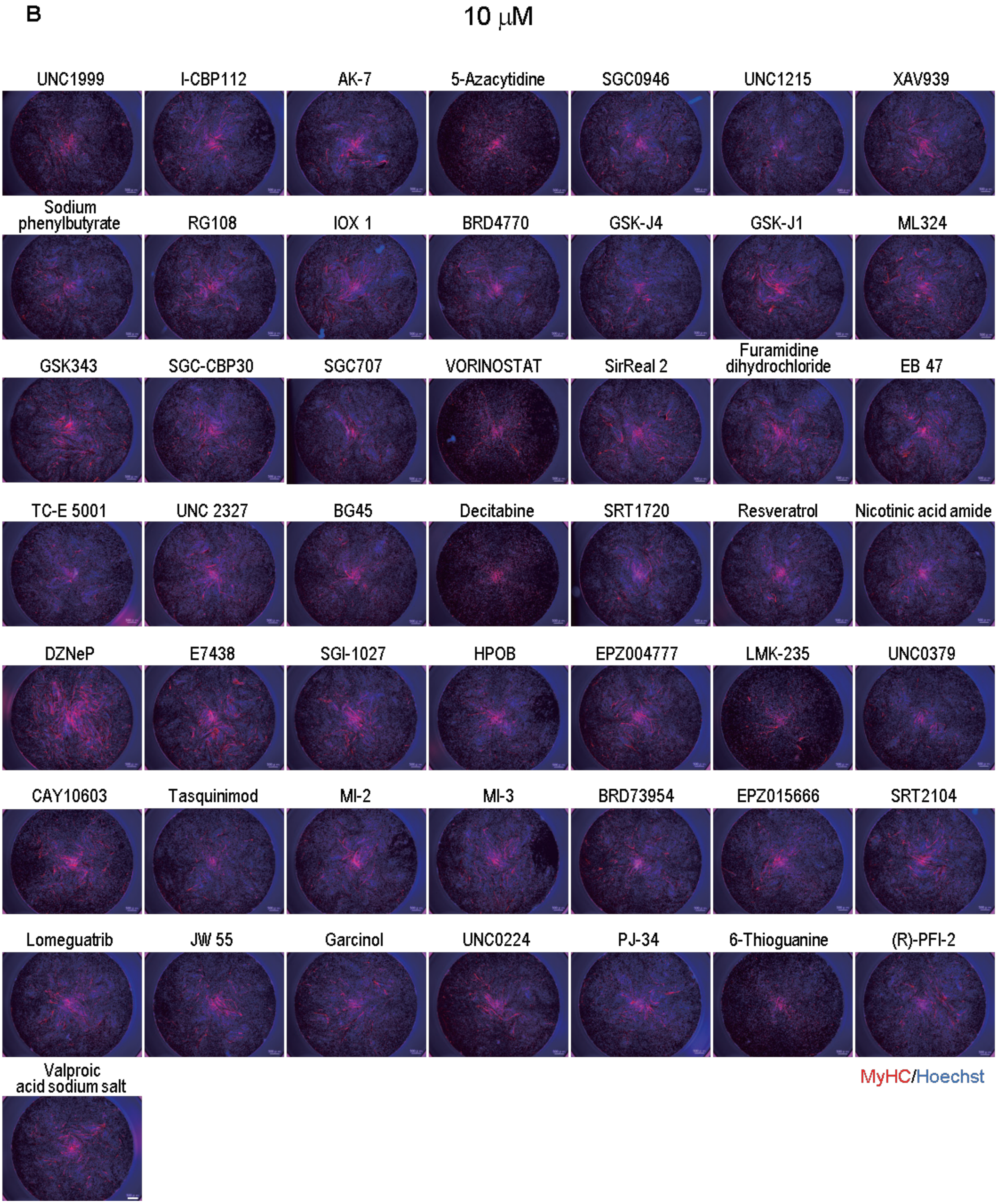

Supplementary Figure S3. Screening of epigenetic drugs that promote myotube formation in *MYOD1*-converted urine-derived cells (UDCs). Immunocytochemistry for myosin heavy chain (MyHC) on the 14th day after myogenic differentiation. Representative images are shown. *MYOD1*-transduced UDCs were treated with epigenetic drugs at 1 μM (A) and 10 μM (B) including 3-deazaneplanocin A hydrochloride (DZNep, histone methyltransferase inhibitor) for initial 3 days after differentiation. Scale bar: 500 μm.

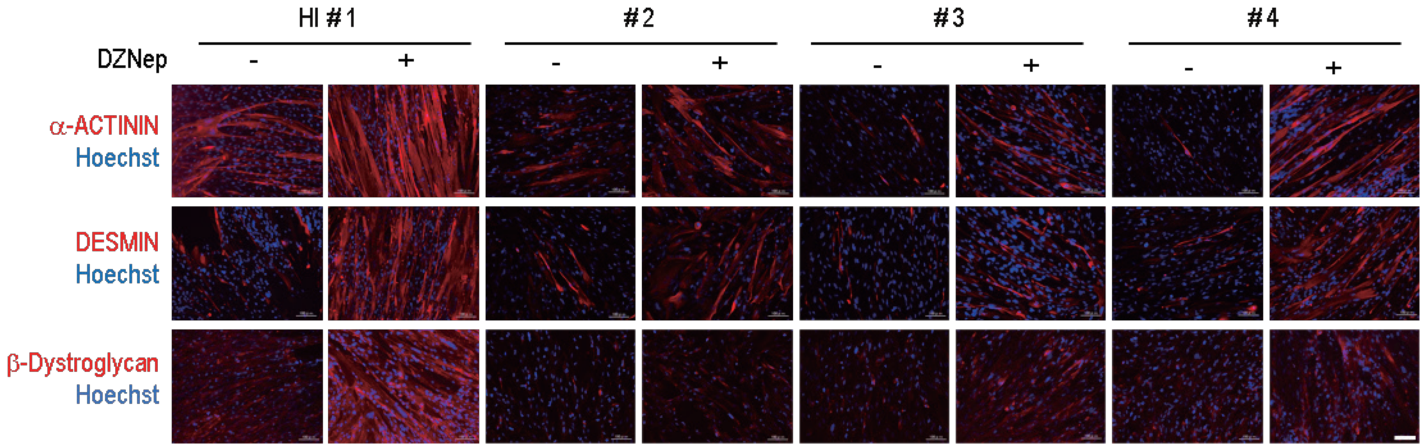

Supplementary Figure S4. Successful myotube differentiation of 3-deazaneplanocin A hydrochloride (DZNep)-treated *MYOD1*-urine-derived cells (UDCs) derived from healthy individuals. Representative images of immunocytochemistry for α-ACTININ, DESMIN, and β-Dystroglycan in *MYOD1*-UDCs from healthy children and adults 14 days after differentiation. UDCs were treated with 1 μM 3-deazaneplanocin A hydrochloride (DZNep) for initial 3 days after differentiation. HI: healthy individual. #1: 8-year-old male; #2: 13-year-old male; #3: 33-year-old male; #4: 38-year-old male. Scale bar: 100 μm.

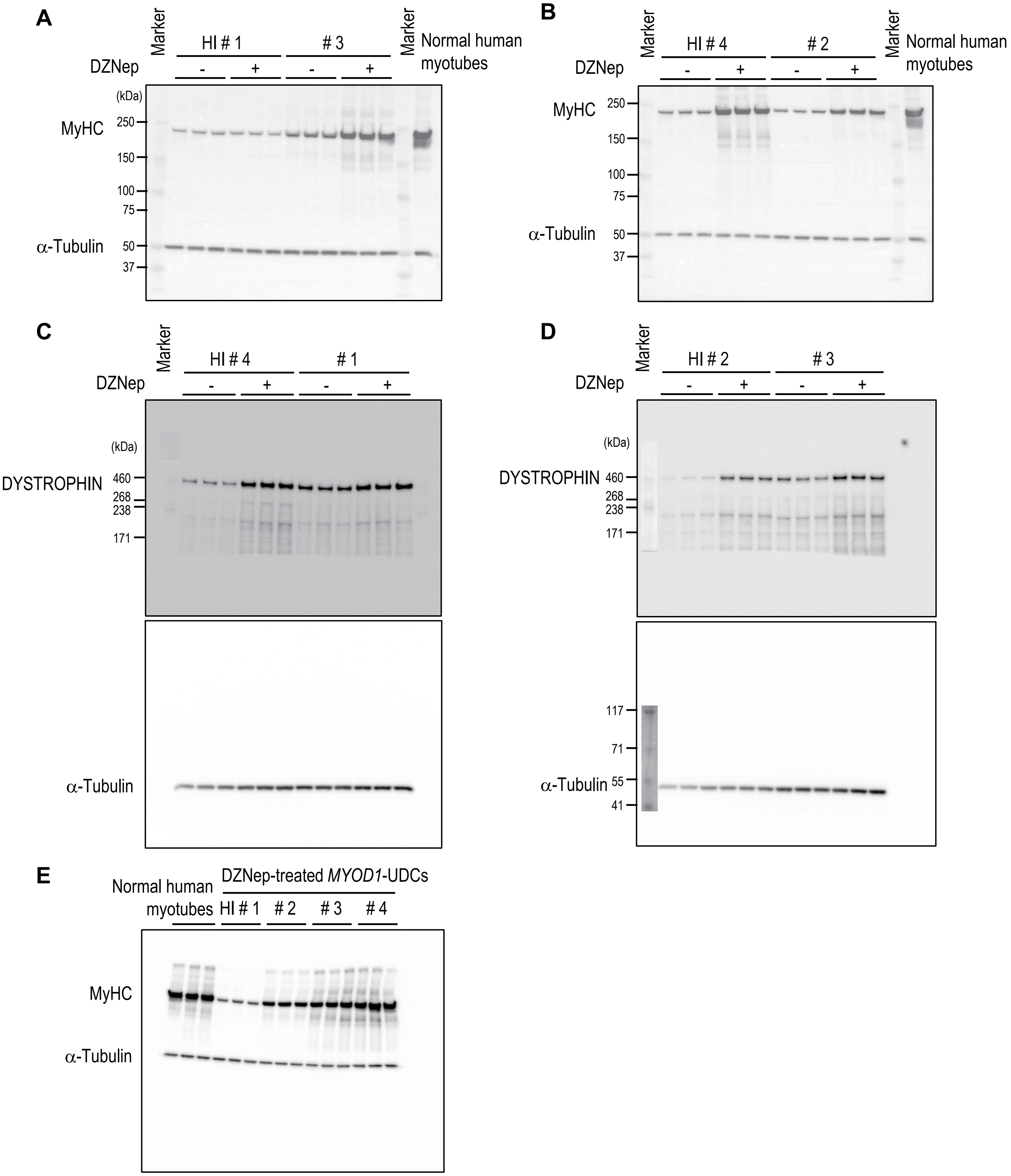

Supplementary Figure S5. Immunoblotting analysis for MyHC and dystrophin using *MYOD1*-converted urine-derived cells (UDCs) from healthy individuals. (A) (B) Images of whole membranes of MyHC staining. An anti-α-Tubulin antibody was used as a loading control. (C) (D) Images of whole membranes of dystrophin staining (upper) and α-Tubulin (lower). (E) Immunoblotting analysis of MyHC in normal human myotubes (Lonza; CC-2580) and DZNep-treated *MYOD1*-UDCs. HI: healthy individual. #1: 8-year-old male; #2: 13-year-old male; #3: 33-year-old male; #4: 38-year-old male.

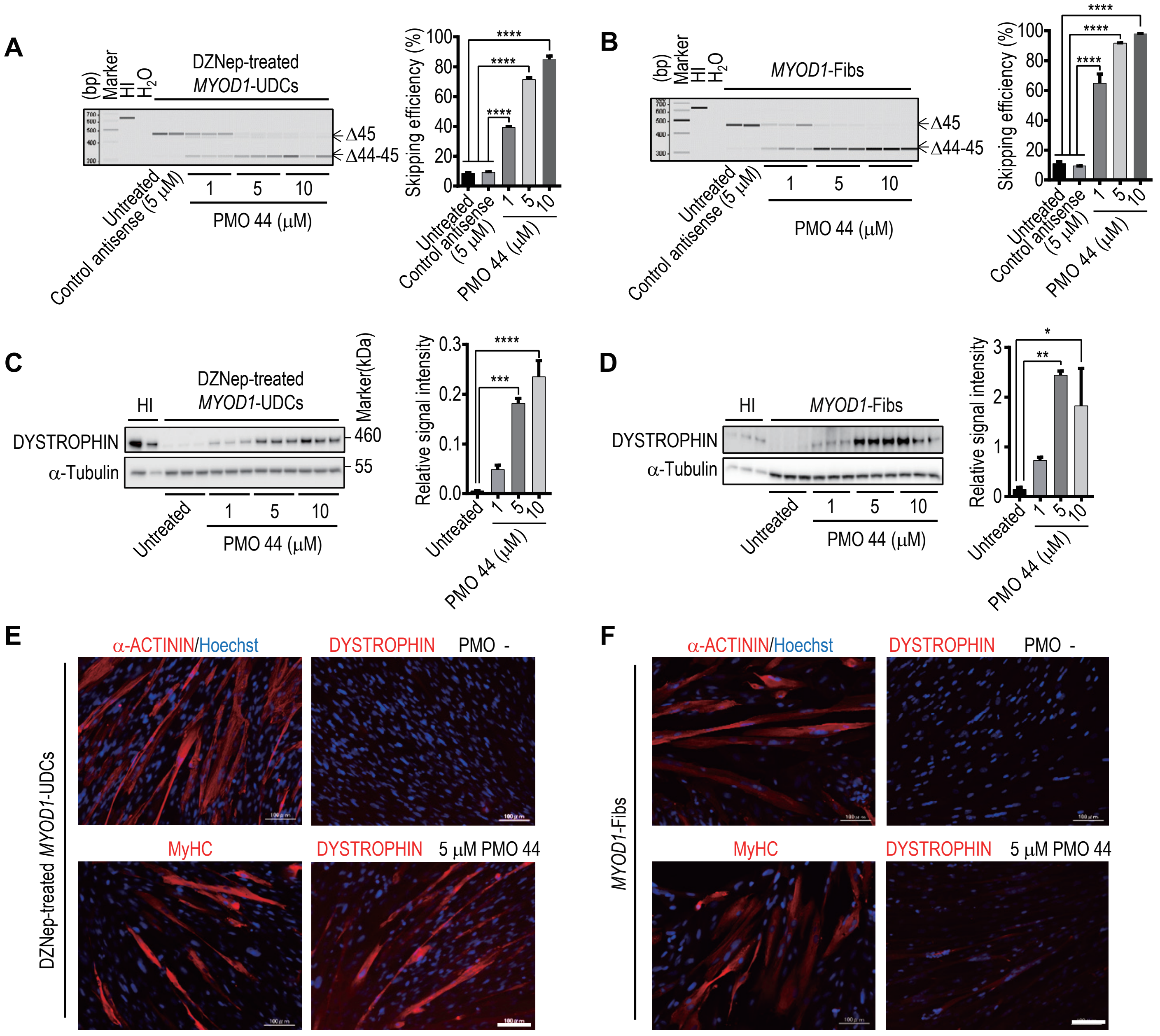

Supplementary Figure S6. Detection of exon-skipping after phosphorodiamidate morpholino oligomer (PMO) treatment in *MYOD1*-urine-derived cells (UDCs) and *MYOD1*-Fibs derived from a Duchenne muscular dystrophy (DMD) patient with a deletion in exon 45. (A, B) Quantification of exon-skipping after PMO treatment evaluated by RT-PCR in DZNep-treated *MYOD1*-UDCs and *MYOD1*-Fibs derived from a DMD patient with exon 45 deletion. The upper bands are unskipped products (Δ45) that remained out of reading frame. The lower bands are exon 44-skipped products (Δ44–45) that restored the open reading frame. Skipping efficiency was calculated as (exon 44-skipped transcript molarity) / (native + exon 44-skipped transcript molarity (marked with arrows)) × 100% using MultiNA. One-way ANOVA followed by Bonferroni's post hoc test was used to compare the skipping efficiencies in patient-derived cells with and without PMO treatment. DZNep-treated *MYOD1*-UDCs and *MYOD1*- Fibs were also treated with control antisense at 5 μM as controls; n = 3, for each. ****P < 0.0001. Data are expressed as mean ± SEM. HI: healthy individual. (C, D) Immunoblotting for dystrophin in DZNep-treated *MYOD1*-UDCs and *MYOD1*-Fibs from the DMD patient after PMO treatment. The relative intensities of the bands normalized to α-Tubulin expression were compared in patient-derived cells with and without PMO treatment by performing a one-way ANOVA followed by a Bonferroni's post hoc test; n = 3, for each; *P < 0.05, **P < 0.01, ***P < 0.001, ****P < 0.0001. (E, F) Immunocytochemistry for α-ACTININ, MyHC, and DYSTROPHIN in DZNep-treated *MYOD1*-UDCs and *MYOD1*-Fibs after PMO treatment. Representative pictures are shown. Scale bar: 100 μm.

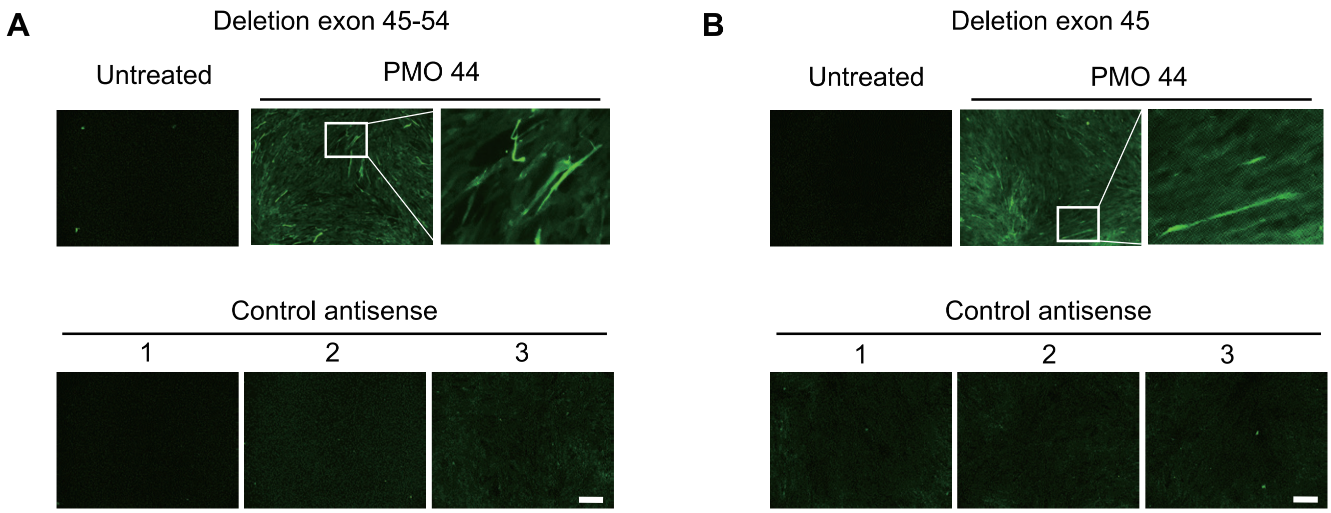

Supplementary Figure S7. Highly efficient and specific screening using a fluorescent microscope. Representative images of immunocytochemistry for DYSTROPHIN (green; scale bar, 300 μm) in 3-deazaneplanocin A hydrochloride (DZNep)-treated *MYOD1*-urine-derived cells (UDCs) from Duchenne muscular dystrophy (DMD) patients (A: patient with exon 45–54 deletion, B: deletion of exon 45, both of which were altered “in frame” by skipping exon 44. PMO44: PMO for skipping exon 44.

Supplementary Table S1. Phosphorodiamidate morpholino oligomer (PMO) sequences and positions.

Target exon	Sequence (5' to 3')	Position	Length (bp)	Reference
44	TGTTCAGCTTCTGTTAGCCACTGA	H44 (+61+84)	24	Wilton, S. D. *et al. Mol Ther*, 2007
50	AACTTCCTCTTTAACAGAAAAGCATA C	H50 (-19-8)	27	Wu, B. *et al. PLoS One*, 2011
51	CTCCAACATCAAGGAAGATGGCATTT CTAG	H51 (+66-95)	30	Cirak, S. *et al. Lancet, 2011*
55	TCTTCCAAAGCAGCCTCT	H55 (+13-30)	18	WO2004/048570

Supplementary Table S2. Antibodies used for FACS analysis.

Primary antibody	Source	Dilution
PE anti-human CD13	BioLegend; 344015	1:20
FITC anti-human CD34	BioLegend; 343603	1:20
APC anti-human CD45	BioLegend; 368511	1:20
FITC anti-human CD73	BioLegend; 344015	1:20
FITC anti-human CD90	BioLegend; 328107	1:20
APC anti-human CD105	BioLegend; 323207	1:10
PE Mouse IgG1, κ Isotype control	BioLegend; 400111	1:20
FITC Mouse IgG1, κ Isotype control	BioLegend; 400109	1:20
FITC Mouse IgG2a, κ Isotype control	BioLegend; 400207	1:20
APC Mouse IgG1, κ Isotype control	BioLegend; 400121	1:10

Supplementary Table S3. RT-PCR primers.

Gene	Forward primer (5’ to 3’)	Reverse primer (5’ to 3’)	Reference
γ-Glutamyl transferase	TGAGCCCAGAAGTGAGAG CAGTTG	ATGTCCACCAGCTCAGAG AGGGT	Yamaguchi, S. et al. Scientific reports,2016
KSP-Cadherin 16	CAAGTCATGAGGTGGTGG TG	TCATCTGTATCCTGGGCC TC	Thedieck, C., et al. Br J Cancer, 2005
Cytokeratin 8	GCCACTACTACACGACCA TCC	GAATCCACCTCCACACTG ACC	Lee, K. M. et al. Laboratory investigation, 2005
AQP1	TAACCCTGCTCGGTCCTT TG	AGTCGTAGATGAGTACAG CCAG	Yamaguchi, S. et al. Scientific reports,2016

Supplementary Table S4. TaqMan primers.

Gene	TaqMan assay ID
MYH2	Hs00430042_m1
DMD	Hs00758098_m1
EIF2B1	Hs00426752_m1
MYOGENIN	Hs01072232_m1
MYOD1	Hs00159528_m1
